# Enhancing antitumor immunogenicity of HPV16-E7 DNA vaccine by fusing DNA encoding E7-antigenic peptide to DNA encoding capsid protein L1 of Bovine papillomavirus

**DOI:** 10.1186/s13578-017-0171-5

**Published:** 2017-08-23

**Authors:** Andrew Yang, Shiwen Peng, Emily Farmer, Qi Zeng, Max A. Cheng, Xiaowu Pang, T. -C. Wu, Chien-Fu Hung

**Affiliations:** 1 Department of Pathology, Johns Hopkins Medical Institutions, Baltimore, MD USA; 2 Department of Oral Pathology, Howard University College of Dentistry, Washington, DC USA; 3 Department of Obstetrics and Gynecology, Johns Hopkins Medical Institutions, Baltimore, MD USA; 4 Department of Molecular Microbiology and Immunology, Johns Hopkins Medical Institutions, Baltimore, MD USA; 5 Department of Oncology, Johns Hopkins Medical Institutions, Baltimore, MD USA; 6 The Johns Hopkins University School of Medicine, CRB II Room 309, 1550 Orleans Street, Baltimore, MD 21231 USA; 7 The Johns Hopkins University School of Medicine, CRB II Room 307, 1550 Orleans Street, Baltimore, MD 21231 USA

**Keywords:** Human papillomavirus 16, E7 antigen, L1 capsid protein, DNA vaccine, Immunotherapy, CD8+ T cell

## Abstract

**Background:**

Human papillomavirus (HPV) has been identified as the primary etiologic factor of cervical cancer, the fourth leading cause of cancer death in females worldwide. We have previously shown that coadministration of DNA encoding L1 capsid protein of Bovine papillomavirus (BPV) can enhance the antigen-specific immune response elicited by a therapeutic HPV16-E7 DNA vaccination. In this study, we sought to generate and evaluate the immunogenicity of a therapeutic HPV16-E7 DNA vaccine that encodes the fusion construct of HPV16-E7 and BPV-L1.

**Results:**

We generated a therapeutic HPV16-E7 DNA vaccine construct, pcDNA3-BPVL1-E7(49-57), encoding the fusion sequence of full-length BPVL1 protein and a murine E7 antigenic epitope, aa49-57. Transfecting 293-D^b^ cells with pcDNA3-BPVL1-E7(49-57) demonstrated that this DNA construct can effectively lead to the presentation of E7 epitope for the activation of E7-specific CD8+ T cells in vitro. Intramuscular vaccination of pcDNA3-BPVL1-E7(49-57) with electroporation generated a stronger E7-specific CD8+ T cell-mediated immune response than coadministration of pcDNA3-BPVL1 and pcDNA3-E7(49-57) in C57BL/6 mice. Furthermore, we observed that the strong E7-specific CD8+ T cell response elicited by pcDNA3-BPVL1-E7(49-57) vaccination translated into potent protective and therapeutic antitumor effects in C57BL/6 mice against HPV16-E7 expressing TC-1 tumor cells. Finally, using antibody depletion experiment, we showed that the antitumor immune response generated by pcDNA3-BPVL1-E7(49-57) is CD8+ T cell dependent, and CD4+ T cell and NK cell independent.

**Conclusion:**

Treatment with fusion construct of BPV-L1 and HPV16-E7 epitope can elicit effective E7-specific antitumor immune response in mice. Due to the potential ability of the fusion DNA construct to also trigger immune responses specific to the L1 protein, the current study serves to support future design of HPV DNA vaccines encoding fusion HPVL1-E6/E7 constructs for the generation of both T cell and B cell mediated immune responses against HPV infections and associated diseases.

## Background

Cervical cancer is the fourth most common cause of cancer death for females worldwide [1, 2]. It is well established that infection with high-risk human papillomavirus (HPV) types, such as HPV-16, is necessary for the progression of cervical cancer [1, 3, 4]. Preventative HPV vaccination can be implemented to inhibit HPV viral infection in patients through the generation of neutralizing antibodies [5]. While prophylactic vaccinations are very successful at preventing new HPV infection, they are unable to treat pre-existing HPV-associated disease in patients whom are already infected. Resultantly, many research efforts have been created to identify methods capable of treating existing HPV-infection and HPV-associated malignancies.

The HPV viral proteins E6 and E7 are the main contributors to the transformation of HPV-infected cells. E6/E7 were shown to enhance proliferation of infected cells [4] and inhibit the function of tumor suppressor genes p53 and pRb [6]. Since these foreign, viral proteins are constantly expressed in infected cells [7], they serve as tumor-specific antigens, allowing for the circumvention of immune tolerance against self-antigens [1], thus providing ideal targets for the development of immunotherapeutic strategies to treat HPV-infection and HPV-associated cancers.

Unlike preventative vaccines, therapeutic HPV vaccines are designed to induce T cell-mediated immune responses to target and destroy infected cells. Particularly, therapeutic DNA vaccines have gained popularity in recent years because they are safe, durable, and easy to manufacture, offering a cost-efficient vaccine regimen [1]. While there are many upsides to using DNA vaccines, they are not capable of amplifying to surrounding cells in vivo, resulting in low immunogenicity. Several strategies were employed to improve DNA vaccine efficacy, such as fusing the DNA sequence of an antigen to that of a MHC pathway homing molecule, or the use of adjuvants (for review see [7]).

The papillomavirus (PV) major capsid protein has been shown to spontaneously self-assemble into virus-like particles (VLPs) upon administration and expression [8, 9]. Additionally, VLPs are non-infectious, as they do not contain any viral genetic material, but can be used to induce neutralizing antibodies or enhance therapeutic antitumor effects against viral proteins [8, 10]. Previously, Bovine papillomavirus (BPV) L1 VLPs have been shown to serve as a strong adjuvant, inducing both humoral and cell-mediated immune response [8, 9, 11, 12] in treated mice. Particularly, in one of our previous studies, we showed that coadministration of a DNA vaccine encoding the fusion protein of calreticulin (CRT) and HPV16-E7 with a DNA plasmid encoding BPVL1 protein generated a stronger E7-specific cytotoxic T lymphocyte (CTL) response than CRT/E7 DNA vaccination alone, suggesting the ability of BPVL1 to effectively stimulate the immune system even when given as DNA instead of VLPs [12].

Thus, in this study, we sought to evaluate the immunogenicity of a therapeutic HPV DNA vaccine encoding a fusion construct of BPVL1 protein and E7aa49-57 antigenic epitope in C57BL/6 mice. Furthermore, we assessed efficacy of this fusion construct in generating antigen-specific antitumor immune effects to protect mice from HPV16-E7 expressing tumor challenge and to treat preexisting TC-1 tumor in mice. Lastly, we determined the immune cell type that is responsible for the observed antitumor responses in vaccinated, TC-1 tumor challenged mice.

## Methods

### Mice

Six- to eight-week-old female C57BL/6 mice were purchased from the National Cancer Institute (Frederick, Maryland, USA) and kept in the oncology animal facility at Johns Hopkins Hospital (Baltimore, Maryland, USA). All animal procedures were performed according to approved protocols by the Johns Hopkins Institutional Animal Care and Committee and in accordance with recommendations for the proper use and care of laboratory animals.

### Cell lines

The HPV16-E6/E7-expressing TC-1 tumor cell line has been previously described [13]. TC-1 cells were cultured in RPMI-1640 medium supplemented with 2 mM glutamine, 1 mM sodium pyruvate, 100 U/mL penicillin, 100 μg/mL streptomycin, 5 × 10^−5^ M β-mercaptoethanol, and 10% fetal bovine serum.

293-D^b^ cell line has been previously described [14]. 293-D^b^ cells were cultured in DMEM medium supplemented with 2 mM glutamine, 1 mM sodium pyruvate, 100 U/mL penicillin, 100 μg/mL streptomycin, and 10% fetal bovine serum.

The murine E7aa49-57 peptide specific CD8+ T cell line has been previously described [15]. The E7-specific CD8+ T cell line were cultured at 37 °C with 5% CO_2_ in RPMI-1640 medium supplemented with 10% FBS, 2 mM l-glutamine, 1 mM sodium pyruvate, 2 mM non-essential amino acids, 100 U/mL penicillin, 100 μg/mL streptomycin, 55 μM 2-Mercaptoethanol, and 25 IU/mL IL-2.

### DNA constructs

To clone pcDNA3-BPVL1, the DNA sequence of BPV1-L1 protein [16] was PCR-amplified by template pshell-BPV (gift from Dr. John Schiller, NIH) and primers (CAGATCTCGAGAATTCACGC and CGACTCTAGAGGCCTACTTCT) and clone into *Xho*I and *Xba*I sites of pcDNA3.1 vector.

To clone pcDNA3-BPVL1-E7(49-57), BPVL1-E7(49-57) was PCR amplified by template pshell-BPV and primers (CAGATCTCGAGAATTCACGC and AAATCTAGATTAAAAGGTTACAATATTGTAATGGGCTCTCTTCTTCTTCTTCTTGGC, which contains the DNA sequence encoding E7aa49-57 epitope) and clone into *Xho*I and *Xba*I sites of pcDNA3.1 vector.

To clone pcDNA3-E7(49-57) (encoding HPV16-E7aa49-57 epitope) minigene, oligos (AATTCATGAGAGCCCATTACAATATTGTAACCTTTTAAA and AGCTTTTAAAAGGTTACAATATTGTAATGGGCTCTCATG) were cloned into *Eco*RI and *Hind*III sites of pcDNA3.1 vector.

### In vitro T cell-activation assay

293-D^b^ cells transfected with pcDNA3-E7(49-57), pcDNA3-BPVL1-E7(49-57), or pcDNA3-BPV-L1 using Lipofectamine 2000. 24 h after the plasmids transfection, the cells were co-incubated with HPV16 E7aa49-57 peptide-specific CD8+ T cell line at an E:T ratio of 1 in the presence of GolgiPlug (1 µL/mL; BD Pharmingen, San Diego, CA) for 20 h at 37 °C. 293-D^b^ cells loaded with HPV16 E7 aa49-57 peptide were used as positive control and 293-D^b^ cells without treatment were used as negative control. After incubation, the CD8+ T cells were collected, washed with FACS wash buffer (PBS containing 0.5% BSA), stained with phycoerythrin-conjugated monoclonal rat antimouse CD8 antibody (BD Pharmingen, San Diego, CA). The cells were then fixed using the Cytofix/Cytoperm kit (BD Pharmingen, San Diego, CA), and intracellularly stained with FITC-conjugated anti-mouse IFN-γ antibody (BD Pharmingen, San Diego, CA). After wash, the cells were acquired with FACSCalibur flow cytometer and analyzed with CELLQuest Pro software (BD Bioscience, Mountain View, CA).

### Electroporation-mediated DNA vaccination

For intramuscular (IM) vaccine injection with electroporation, (1) 20 μg of empty pcDNA3 vector, (2) 10 μg of pcDNA3-E7(49-57) + 10 μg of empty pcDNA3 vector, (3) 10 μg of pcDNA3-BPVL1 + 10 μg of pcDNA3-E7(49-57), or (4) 10 μg of pcDNA3-BPVL1-E7(49-57) + 10 μg of empty pcDNA3 vector were prepared and injected in the tibialis muscle of the shaved hind leg of mice followed by electroporation with an ECM830 Square Wave Electroporation System (BTX Harvard Apparatus company, Holliston, MA, USA). Booster vaccination(s) was administered using the same dose and regimen as the priming vaccination.

### Intracellular cytokine staining with flow cytometric analysis to detect IFN-γ secretion by E7-specific CD8+ T cells

Splenocytes from vaccinated groups were collected and incubated for 20 h with 1 μg/mL of MHC class I restricted E7 peptide (aa49-57, RAHYNIVTF). 1 µL/mL of GolgiPlug was added 6 h before the cells were harvested. Cells were then washed once in FACScan buffer and stained with PE-conjugated monoclonal rat antimouse CD8 antibody. The cells were then fixed using the Cytofix/Cytoperm kit. Intracellular IFN-γ was stained with FITC-conjugated anti-mouse IFN-γ antibody. After wash, the cells were acquired with FACSCalibur flow cytometer and analyzed with CELLQuest Pro software.

### In vivo tumor protection experiments

C57BL/6 mice (5 per group) were vaccinated with (1) 20 μg of empty pcDNA3 vector, (2) 10 μg of pcDNA3-E7(49-57) + 10 μg of empty pcDNA3 vector, (3) 10 μg of pcDNA3-BPVL1 + 10 μg of pcDNA3-E7(49-57), or (4) 10 μg of pcDNA3-BPVL1-E7(49-57) + 10 μg of empty pcDNA3 vector via IM injection with electroporation followed by a booster vaccination 1 week later. One week after the last vaccination, mice were challenged subcutaneously (SC) with 5 × 10^4^ TC-1 cells/mice. TC-1 cells challenged mice without treatment were used as control. Survival of mice was followed for 100 days after tumor challenge. Tumor growth was determined by direct palpation once a week. When the tumor growth exceeds 1.5 cm in diameter, the mice were considered to have died from tumor burden, and subsequently euthanized.

### In vivo tumor treatment experiment

C57BL/6 mice (5 per group) were challenged SC with 5 × 10^4^ TC-1 cells/mouse. Two days after tumor challenge, mice were primed IM with (1) 20 μg of empty pcDNA3 vector, (2) 10 μg of pcDNA3-E7(49-57) + 10 μg of empty pcDNA3 vector, (3) 10 μg of pcDNA3-BPVL1 + 10 μg of pcDNA3-E7(49-57), or (4) 10 μg of pcDNA3-BPVL1-E7(49-57) + 10 μg of empty pcDNA3 vector. Mice were boosted with the same dose and regimen 7 days later. Tumor growth was determined by direct palpation once a week and the formation of detectable tumor was noted. When the tumor growth exceeds 1.5 cm in diameter, the mice were considered to have died from tumor burden, and subsequently euthanized.

### In vivo antibody depletion experiments

C57BL/6 mice (5 per group) were primed IM with 10 μg/mouse of pcDNA3-BPVL1-E7(49-57) and boosted with the same dose and regimen 7 days later. Mice underwent antibody depletion of CD4, CD8 or natural killer cells via injection of 100 μg purified rat anti-mouse monoclonal antibodies against CD4 (clone GK1.5), CD8a (clone 2.43), or NK1.1 (clone PK136), 1 day after the last vaccination. The depletion efficacy of the antibody administrations are >90% based on our previous studies [17–19]. The mice were injected again with the same amount of antibodies every other day for an additional three times for the first week and then once every week afterward. All depletion antibodies were purchased from BioXCell (West Lebanon, NH). Seven days after the last vaccination, mice were challenged SC with 5 × 10^4^ TC-1 cells/mouse. Tumor bearing, pcDNA3-BPVL1-E7 vaccinated mice without antibody depletion and untreated tumor bearing mice were used as controls. After tumor challenge, tumor growth was determined by direct palpation once a week and the formation of detectable tumor was noted. When the tumor growth exceeds 1.5 cm in diameter, the mice were considered to have died from tumor burden, and subsequently euthanized.

### Statistical analysis

Data expressed as mean ± standard deviation (SD) are representative of a minimum of two separate experiments. Comparisons between individual data points were made by two-tailed student’s *t* tests. Tumor free or survival distributions for mice in different groups were compared through Kaplan–Meier curves and Log-rank tests. A *p* < 0.05 was considered statistically significant.

## Results

### Presentation of HPV16 E7aa49-57 by 293-D^b^ cells transfected with pcDNA3-BPVL1-E7(49-57)

**Fig. 1:**
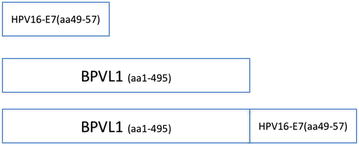
DNA construct of pcDNA3-E7(49-57), pcDNA3-BPVL1, and pcDNA3-BPVL1-E7(49-57). The DNA sequence of HPV16 E7 CTL epitope (aa49-57) and the full-length sequence of BPV1 L1 protein were individually cloned into the pcDNA3 plasmid vector to generate pcDNA3-E7(49-57) and pcDNA3-BPVL1, respectively. The DNA sequence of HPV16-E7aa49-57 epitope was fused to DNA sequence of the full-length BPV1 L1 protein at the C-terminus and cloned into the pcDNA3 plasmid vector to generate the pcDNA3-BPVL1-E7(49-57) fusion construct

**Fig. 2:**
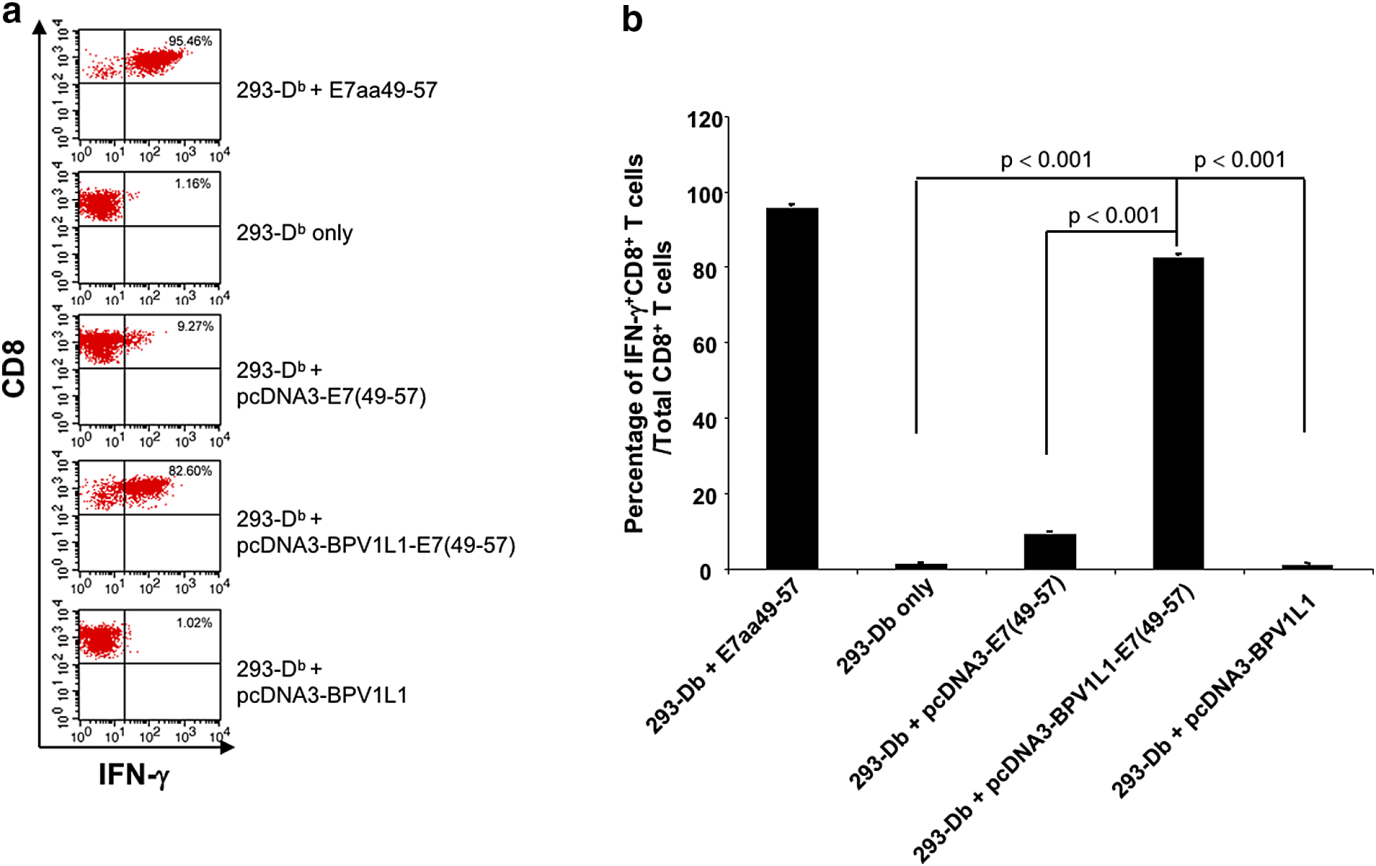
Presentation of HPV16 E7 antigen by 293-D^b^ cells transfected with various plasmid constructs. HPV16-E7aa49-57 peptide loaded or pcDNA3-E7(49-57), pcDNA3-BPVL1-E7(49-57) or pcDNA3-BPVL1 transfected 293-D^b^ cells were co-incubated with HPV16 E7aa49-47 peptide-specific CD8+ T cell line (E:T ratio at 1:1). Untreated 293-D^b^ cells were used as negative control. After co-incubation, the E7-specific CD8+ T cells were stained for surface CD8 and intracellular IFN-γ and acquired with FACSCalibur flow cytometer. The data was analyzed using CellQuest software. **a** Representative flow cytometry analysis. **b** Bar graph summary of the flow analysis

The pcDNA3-BPVL1-E7(49-57) fusion construct was designed by fusing the HPV16 E7 CTL epitope (aa49-57) to the C-terminus of the BPVL1 sequence (Fig. 1). To evaluate the presentation of E7 epitope by this fusion construct, we transfected 293-D^b^ cells with pcDNA3-E7(49-57), pcDNA3-BPVL1-E7(49-57) or pcDNA3-BPVL1, and co-cultured transfected 293-D^b^ cells with an E7-specific CD8+ T cell line. Using E7aa49-57 peptide pulsed 293-D^b^ cells and 293-D^b^ cells without treatment as controls, the presence of activated E7-specific CTLs after co-culturing with the 293-D^b^ cells were assessed using surface CD8 and intracellular IFNγ staining, followed by flow cytometry analysis (Fig. 2a). As shown in Fig. 2a, b, 293-D^b^ cells pulsed with E7(aa49-57) epitope generated the strongest E7-specific CD8+ T cell response. Additionally, 293-D^b^ cells that were transfected with pcDNA3-BPVL1-E7(49-57) or pcDNA3-E7(49-57) also showed a strong and weak E7-specific CD8+ T cell response, respectively, while 293-D^b^ cells alone, or 293-D^b^ cells transfected with pcDNA3-BPVL1 plasmid showed negligible E7-specific T cell responses (Fig. 2b).

### pcDNA3-BPVL1-E7(49-57) vaccination induced potent E7-specific immune response in vivo

**Fig. 3:**
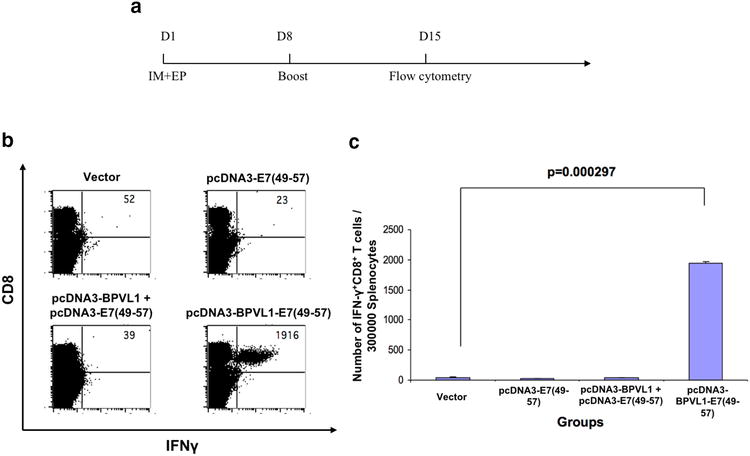
Analysis of E7-antigen-specific immune response generated by pcDNA3-BPVL1-E7(49-57) vaccination. **a** Schema of experiment. Briefly, 6–8 week old female C57BL/6 mice (5 per group) were vaccinated IM with (1) 20 μg of empty pcDNA3 vector, (2) 10 μg of pcDNA3-E7(49-57) + 10 μg of empty pcDNA3 vector, (3) 10 μg of pcDNA3-BPVL1 + 10 μg of pcDNA3-E7(49-57), or (4) 10 μg of pcDNA3-BPVL1-E7(49-57) + 10 μg of empty pcDNA3 vector, followed by electroporation on day 1. Mice were boosted with the same regimen on day 7. **b**, **c** Seven days after the last vaccination, splenocytes from the mice were collected and stimulated with HPV16 E7aa49-57 peptide in the presence of GolgiPlug, stained for surface CD8 and intracellular IFN-γ, and analyzed using flow cytometry. **b** Representative flow cytometry image for the amount of IFNγ + CD8+ T cells among splenocytes. **c** *Bar graph* summary of flow cytometry analysis

To evaluate the immunogenicity of the fusion pcDNA3-BPVL1-E7(49-57) in vivo, mice were vaccinated IM in the hind leg with either pcDNA3-BPVL1-E7(49-57), pcDNA3-BPVL1 + pcDNA3-E7(49-57), pcDNA3-E7(49-57), or empty pcDNA3 vector, followed by electroporation. Mice then received the same regimen 1 week later. Seven days after the last vaccination, splenocytes were collected, stimulated with HPV16-E7aa49-57 peptide and stained for surface CD8 and intracellular IFN-γ (Fig. 3a). The mice that received vaccination with empty vector, pcDNA3-E7(49-57), or pcDNA3-BPVL1 + pcDNA3-E7(49-57) followed by electroporation had a weak E7-specific CD8+ T cell response; however, mice that were vaccinated with pcDNA3-BPVL1-E7(49-57) had a robust E7-specific CD8+ T cell response (Fig. 3b, c). This demonstrates that vaccination with the pcDNA3-BPVL1-E7 vaccine were able to elicit a potent antigen-specific immune in treated mice.

### Vaccination with pcDNA3-BPVL1-E7(49-57) generated potent protective effect against TC-1 tumor challenge in vivo

**Fig. 4:**
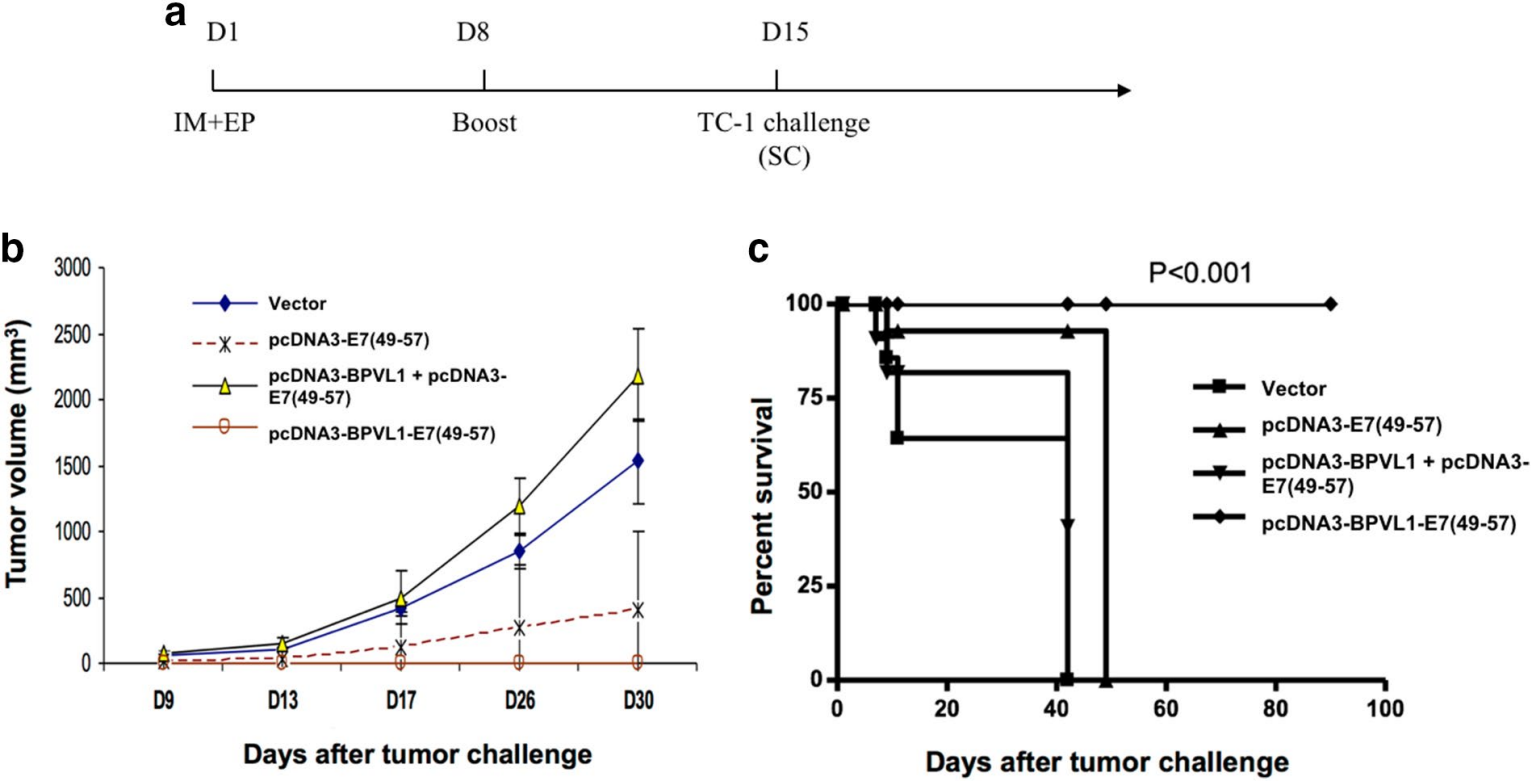
In vivo tumor protection experiments. **a** Schema of experiment. Briefly, 6–8 week old female C57BL/6 mice (5 per group) were vaccinated IM with (1) 20 μg of empty pcDNA3 vector, (2) 10 μg of pcDNA3-E7(49-57) + 10 μg of empty pcDNA3 vector, (3) 10 μg of pcDNA3-BPVL1 + 10 μg of pcDNA3-E7(49-57), or (4) 10 μg of pcDNA3-BPVL1-E7(49-57) + 10 μg of empty pcDNA3 vector, followed by electroporation on day 1. Mice received the same regimen 7 days later. Eight days after the last vaccination, on day 15, mice were challenged SC with 5 × 10^4^ TC-1 tumor cells, including five naïve mice that did not receive any vaccination. **b** Line graph depicting the change in tumor volume in TC-1 challenged mice over 30 days. **c** Kaplan–Meier survival plot of TC-1 tumor-bearing mice was recorded over 100 days

In order to test whether pcDNA3-BPVL1-E7(49-57) vaccine can generate protective antitumor effect against tumor challenge in mice, mice were vaccinated with the same regimen used in Fig. 3. Eight days after the last vaccination, mice were challenged SC with TC-1 tumor cells, with unvaccinated, TC-1 tumor challenged mice as control (Fig. 4a). After tumor challenge, tumor growth and survival of mice were monitored. No observable tumor formation was observed in mice immunized with pcDNA3-BPVL1-E7(49-57) as compared to mice in other treatment groups (Fig. 4b). Furthermore, all mice immunized with pcDNA3-BPVL1-E7(49-57) survived over 100-days after tumor challenge, while mice in other treatment groups all died within 50 days after tumor challenge (Fig. 4c). These data suggest that pcDNA3-BPVL1-E7(49-57) vaccine is able to generate potent immune responses that protect vaccinated mice from TC-1 tumor challenge.

### pcDNA3-BPVL1-E7(49-57) vaccination generated potent therapeutic antitumor effects in TC-1 tumor challenged mice

**Fig. 5:**
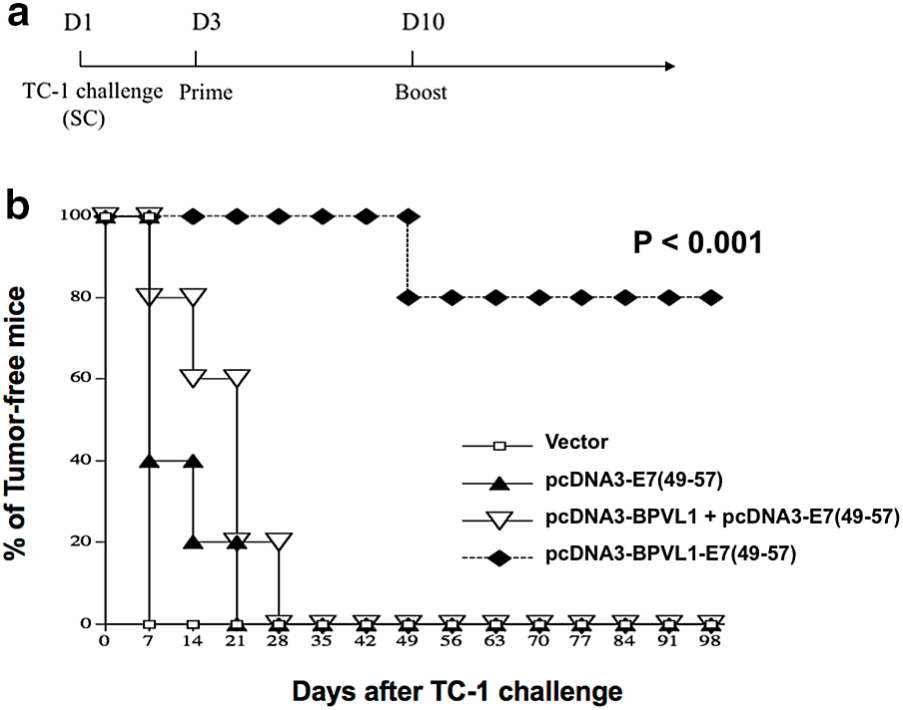
In vivo tumor treatment experiments. **a** Schema of experiment. Briefly, 6–8 week old female C57BL/6 mice (5 per group) were challenged SC with 5 × 10^4^ TC-1 tumor cells on day 1. Three days after tumor challenge, mice were primed IM with (1) 20 μg of empty pcDNA3 vector, (2) 10 μg of pcDNA3-E7(49-57) + 10 μg of empty pcDNA3 vector, (3) 10 μg of pcDNA3-BPVL1 + 10 μg of pcDNA3-E7(49-57), or (4) 10 μg of pcDNA3-BPVL1-E7(49-57) + 10 μg of empty pcDNA3 vector, with electroporation. Mice were then boosted with the same regimen 1 week later, on day 10. **b** The formation of detectable tumor in mice was monitored once a week via palpation for 100 days

After demonstrating that pcDNA3-BPVL1-E7(49-57) could protect mice from TC-1 tumor challenge when administered prophylactically, we sought to determine whether pcDNA3-BPVL1-E7(49-57) could be used therapeutically to treat mice bearing TC-1 tumor. Mice were challenged with TC-1 tumor cells via SC injection on day 1. Mice then received IM injection of pcDNA3-BPVL1-E7(49-57), pcDNA3-BPVL1 + pcDNA3-E7(49-57), pcDNA3-E7(49-57), or empty vector on day 3, and were boosted with the same regimen 7 days later (Fig. 5a). Formation of detectable tumor mass was then monitored once a week for 100 days following last vaccination. Eighty percent of the pcDNA3-BPVL1-E7(49-57) vaccinated mice did not develop detectable tumor for at least 100 days after tumor challenge, while all other mice grew tumors by day 28 (Fig. 5b). Thus, we believe that the pcDNA3-BPVL1-E7(49-57) vaccine has the potential to be used therapeutically to control the growth of tumor.

### Antigen-specific antitumor immune response elicited by pcDNA3-BPVL1-E7(49-57) vaccine is CD8+ T cell dependent

**Fig. 6:**
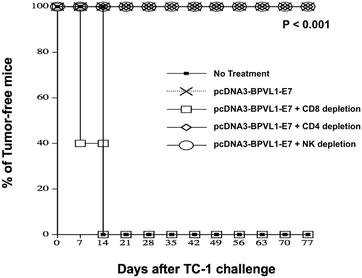
In vivo antibody depletion experiments. Six to eight week old female C57BL/6 mice (5 per group) were primed with 10 µg of pcDNA3-BPVL1-E7(49-57) vaccine on day 1. Mice were boosted with the same dose and regimen 7 days later. One day after last vaccination, mice were injected with anti-CD4, -CD8, or -NK1.1 antibody to deplete the respective cell type. The mice were injected with the same antibodies every other day, for a total three times during the first week, then one time each week using the same regimen for the next 11 weeks. One week after last vaccination, mice were challenged SC with 5 × 10^4^ TC-1 tumor cells. One group of treated tumor-bearing mice without antibody depletion, and one group of untreated tumor-bearing mice were used as controls. The formation of detectable tumor in mice was monitored once a week via palpation for 100 days

In order to determine the involvement of various immune cell types in the antigen-specific antitumor immune response elicited by pcDNA3-BPVL1-E7(49-57) vaccination, we performed in vivo immune cell depletion using antibodies. Mice were primed with pcDNA3-BPVL1-E7(49-57) vaccine and boosted with the same regimen 7 days later. One day after last vaccination, mice began to receive antibody depletion of CD4, CD8, or NK cells, for a total of three times during the first week, then one time per week with the same regimen for the next 11 weeks. Seven days after the vaccination boost, on day 21, mice were challenged SC with TC-1 tumor cells. pcDNA3-BPVL1-E7(49-57) vaccinated, tumor-challenged mice without antibody depletion and untreated tumor-challenged mice were used as controls. As shown in Fig. 6, 100% of mice that did not receive antibody depletion or mice that received CD4+ T cell or NK cell depletion remained tumor-free for at least 11 weeks after tumor challenge. Conversely, all mice that received CD8+ T cell depletion grew detectable tumors by week 2, comparable to that of untreated mice. These data indicate that the antitumor immune response elicited by pcDNA3-BPVL1-E7(49-57) vaccination is CD8+ T cell dependent.

## Discussion

In this study, we found that our vaccine, pcDNA3-BPVL1-E7(49-57), could elicit a stronger E7-specific CD8+ T cell-mediated immune response than pcDNA3-E7(49-57) alone or in combination with pcDNA3-BPVL1. We also found that vaccination with pcDNA3-BPVL1-E7(49-57) could elicit potent protective and therapeutic antitumor responses against HPV16-E7-expressing TC-1 tumor model in mice. Lastly, we show that the pcDNA3-BPVL1-E7(49-57)-induced antitumor immune response was CD8+ T cell dependent.

We have previously generated several DNA constructs fusing HPV-E6/E7 antigens to molecules such as LAMP-1 [20], heat shock protein 70 [21], Flt3-ligand [22], translocation domain of Pseudomonas aeruginosa exotoxin A [22], VP22 [23, 24], CRT [17, 25], and gamma-tubulin [26], in order to enhance the immunogenicity of therapeutic HPV DNA vaccines. Most of these fusion DNA vaccine constructs primarily enhance either CD8+ T cell or CD4+ T cells against HPV-E6/E7 antigens. More recently, we examined a strategy to enhance the immunogenicity of a therapeutic HPV DNA vaccine encoding the fusion construct CRT-E7 by coadministration with DNA encoding the L1 or L2 protein of papillomavirus. We demonstrated that not only did the coadministration of CRT/E7 DNA with L1 or L2 DNA enhance the CD8+ T cell-mediated E7-specific antitumor response, it also induced strong L1/L2-specific CD4+ T cell and antibody responses [12]. In this study, we showed that the fusion BPVL1-E7(49-57) DNA construct could similarly enhance E7-specific CD8+ T cell-mediated antitumor immune responses in C57BL/6 mice. We expect the BPVL1-E7(49-57) fusion DNA will also generate BPVL1-specific T cell and B cell responses, leading to neutralizing antibody production. Future studies are warranted to confirm the ability of papillomavirus L1-E7 fusion DNA construct to serve as a potential hybrid therapeutic and preventative vaccine.

There are several potential mechanisms that contribute to the enhance immunogenicity of pcDNA3-BPVL1-E7(49-57) DNA vaccine observed in the current study. We have previously observed that coadministration of DNA encoding BPVL1 enhances the immunogenicity of CRT/E7 DNA vaccine by increasing CD4+ T cell responses that are known to assist in the generation of CD8+ T cell response [12], which serve as the basis for the design of a fusion BPVL1-E7 DNA vaccine. Additionally, multiple studies have tested the conjugation of E7 to L1 VLPs as a method to enhance the E7 immune response. It has been shown that intact HPV-L1 VLPs can interact with DCs to directly induce potent adaptive immune responses in the absence of adjuvants [27], potentially through a TLR-MyD88 pathway-dependent manner [28]. On the other hand, it is also possible that the DNA sequence of BPVL1 encoded in the plasmid is recognized by DNA sequence sensors such as STING or TLR-9 to exert adjuvant effects (for review see [29]). However, the enhanced immunogenicity was not observed in the co-administration of pcDNA3-E7(49-57) and pcDNA3-BPVL1 as compared to single administration of pcDNA3-BPVL1-E7(49-57) (Figs. 3, 4 and 5), suggesting that DNA sensing is not the major contributor to the enhanced immunogenicity for the fusion DNA construct observed in the current study. Importantly, previous studies have demonstrated that fusing the DNA sequence of BPVL1 to that of E7aa49-57 can generate the in vitro formation of BPVL1-E7(49-57) VLPs, which serve as a carrier to target the E7 antigen to DCs and enhance both the MHC class I and class II pathways, resulting in potent E7-specific CTL response and antibody response against BPVL1 [8, 9]. Similar results were also observed when the BPV-L1 is conjugated to the HIV-1 gp41 protein, resulting in the generation of potent antibody response specific to gp41 [11, 30]. Thus, the enhanced immunogenicity observed from the vaccination with pcDNA3-BPVL1-E7(49-57) fusion construct may be due to more than just the adjuvant effects observed from our previous study [12]. Based on the in vitro antigen presentation experiment, pcDNA3-BPVL1-E7(49-57) transfected 293-D^b^ cells generated a stronger E7-specific CD8+ T cell activation than pcDNA3-E7(49-57) transfected 293-D^b^ cells, suggesting that fusing E7(49-57) to BPVL1 could enhance the processing and presentation of the E7 peptide antigen as compared to E7(49-57) alone (Fig. 2). Thus, the increased immunogenicity of pcDNA3-BPVL1-E7(49-57) observed in mice may be caused by a consortium of enhanced antigen processing and presentation as well as the stimulation of innate immunity rather than one single mechanism. Since it has been well-established that the papillomavirus major capsid protein L1 is able to self-assemble into VLPs when expressed in both eukaryotic and prokaryotic systems [8, 9], it is possible that vaccination with BPVL1-E7(49-57) DNA can result in the in vivo assembly of BPVL1-E7 VLPs, and target the antigens to DCs for the generation of the observed, enhanced E7-specific CTL response.

Of note, while we hypothesize that the inclusion of BPVL1 will lead to the generation of CD4+ T cells to assist in elicitation of E7-specific CD8+ T cell response based on our previous publication [12], we showed that CD4 depletion following immunization did not abolish the observed protective antitumor effects (Fig. 6). We have previously demonstrated that depletion of CD4+ T cells prior to the administration of therapeutic vaccine will significantly reduce the resultant CD8+ T cell response elicited by vaccination [18]. However, in the current study, CD4 depletion was not performed until after the immunization had been completed. It has been well demonstrated that CD8+ T cell response is the main contributor for the control of TC-1 tumor model [17]. Thus, once the E7-specific CD8+ T cell response has been generated, the CD4+ T cells do not appear to be necessary for the subsequent protection of tumor formation.

The possibility that the BPVL1-E7 fusion DNA design may elicit E7-specific CD8+ T cell response as well as L1-specific T cell and B cell responses as a single DNA construct holds significant translational values. While the currently available HPV-L1 VLP-based prophylactic vaccines, such as Cervarix, Gardasil, and Gardasil-9, are highly effective, their use in many developing countries are impeded by the technically demanding process and high costs associated with VLP vaccine production and the difficulty of storing and maintaining the VLP vaccines (for review see [31–33]). Kwak et al. has previously suggested the potential of HPVL1 DNA vaccines to generate strong L1-specific antibody response comparable to that generated by Gardasil in vivo [34]. As such, DNA vaccines capable of generating effective neutralizing antibody against HPV, in addition to the generation of HPV-specific T cell-mediated immunity, may significantly increase the availability of HPV immunization. Since the DNA construct designed in the current study, pcDNA3-BPVL1-E7(49-57), encodes the L1 protein of BPV and the murine antigenic epitope of HPV16-E7, it is likely to have minimal benefit as a clinical therapeutic vaccine candidate, but merely serves as a proof of concept for the generation of hybrid therapeutic and prophylactic HPV vaccine. Future studies are warranted for the generation and evaluation of a HPV DNA vaccine construct encoding HPVL1 or L2 protein fused to the human antigenic epitopes or full-length proteins of HPV-E6/E7, for the generation of neutralizing antibodies against the L1/L2 capsid proteins as well as the E6/E7-specific CTL responses for the protection and treatment of HPV-associated infections and diseases.

## Conclusions

In summary, we demonstrated that treatment with fusion construct of BPV-L1 and HPV16-E7 epitope can elicit effective E7-specific antitumor immune response in mice. Due to the potential ability of the fusion DNA construct to also trigger immune responses specific to the L1 protein, the current study serves to support future design of HPV DNA vaccines encoding fusion HPVL1-E6/E7 constructs for the generation of both T cell and B cell mediated immune responses against HPV infections and associated diseases.

## Abbreviations

HPV: human papillomavirus
E6: HPV early protein 6
E7: HPV early protein 7
BPV: Bovine papillomavirus
BPVL1: Bovine papillomavirus late protein 1
VLP: virus like particle
IM: intramuscular
FITC: fluorescein isothiocyanate
IFN-γ: interferon gamma
CTL: cytotoxic T lymphocyte
SC: subcutaneous
NK: natural killer
